# Supracondylar Humerus Fractures in Children With Vascular Compromise: A Report of Two Cases and Review of Literature

**DOI:** 10.7759/cureus.109999

**Published:** 2026-05-31

**Authors:** Majid Kazemtash, Carola Hecking, Clemens Höflich, Sofia Stefanidou, Thomas Schmandra

**Affiliations:** 1 Vascular Surgery, Sana Klinikum Offenbach, Offenbach am Main, DEU

**Keywords:** anticoagulation, pediatric trauma, rivaroxaban, supracondylar humerus fracture, vascular injury

## Abstract

Supracondylar humerus fractures are the most common pediatric elbow fractures and may rarely be associated with vascular compromise. We present two pediatric cases of displaced left supracondylar humerus fractures complicated by arterial injury. A four-year-old boy developed absent radial and ulnar pulses following fracture fixation and underwent urgent thrombectomy with Fogarty catheterization using a Fogarty arterial embolectomy catheter (Edwards Lifesciences, Irvine, CA, USA), resulting in immediate restoration of distal perfusion. An 11-year-old girl presented with brachial artery entrapment within the fracture site and was successfully treated with surgical release and vascular repair. Both patients were managed with external fixation (DePuy Synthes, West Chester, PA, USA) and received postoperative weight-adjusted rivaroxaban (Xarelto®; Bayer AG, Leverkusen, Germany) as part of individualized postoperative management for three months. At follow-up, both patients demonstrated complete recovery of hand function with intact distal perfusion and no neurological deficits. These cases highlight the importance of early recognition of vascular compromise, prompt surgical intervention, and interdisciplinary management in preventing ischemic complications and optimizing functional recovery. However, evidence regarding postoperative anticoagulation in pediatric vascular trauma remains limited, and standardized protocols are currently lacking.

## Introduction

Supracondylar humerus fractures are the most common pediatric elbow fractures, accounting for approximately 60% of elbow injuries in children, and most frequently occur between the ages of five and eight years [1,2]. Displaced fractures, particularly Gartland type III and IV injuries, are associated with an increased risk of neurovascular compromise because of hyperextension trauma, posterior fragment displacement, arterial entrapment, intimal injury, thrombosis, or vasospasm [3-5]. The brachial artery is the vessel most commonly affected due to its close anatomical relationship to the distal humerus and antecubital fossa structures [3-5].

Prompt recognition of vascular compromise is essential to prevent limb ischemia and long-term functional impairment. Clinical neurovascular assessment should include palpation of the brachial, radial, and ulnar pulses, evaluation of hand color and temperature, capillary refill time, Doppler examination when available, and detailed motor-sensory assessment [6,7]. A clinically important distinction should be made between the “pink pulseless hand” and the “pale pulseless hand.” A pale, cold, pulseless extremity suggests critical ischemia and generally requires immediate fracture reduction and urgent vascular exploration, whereas a pink pulseless hand with preserved perfusion may occasionally permit close observation after reduction [6,7].

Initial management focuses on urgent fracture reduction and restoration of distal perfusion. Although Doppler ultrasonography and CT angiography may assist in selected cases, advanced vascular imaging should not delay operative intervention in patients with clear clinical signs of limb-threatening vascular compromise [6-8]. Treatment depends on fracture severity and associated neurovascular injury. Non-displaced fractures are usually managed conservatively with immobilization, whereas displaced fractures commonly require closed or open reduction with Kirschner wire fixation [1-3]. However, in selected cases with associated vascular injury, external fixation may provide stable fracture alignment while simultaneously facilitating unrestricted vascular access during exploration and repair.

Vascular exploration may be necessary in cases of persistent ischemia, absent pulses after reduction, arterial entrapment, thrombosis, or suspected arterial injury [4,5]. Surgical options include thrombectomy, primary arterial repair, release of entrapped vessels, or interposition grafting, depending on intraoperative findings. Postoperative management includes close neurovascular monitoring, pain control, physiotherapy, and individualized anticoagulation strategies in selected vascular repair cases. However, evidence regarding postoperative anticoagulation in pediatric vascular trauma remains limited, and standardized treatment protocols are currently lacking.

We present two pediatric cases of displaced supracondylar humerus fractures complicated by vascular compromise requiring urgent vascular intervention. Both patients underwent external fixation combined with vascular exploration and postoperative anticoagulation with favorable functional and vascular outcomes.

## Case presentation

Case 1

**Figure 1:**
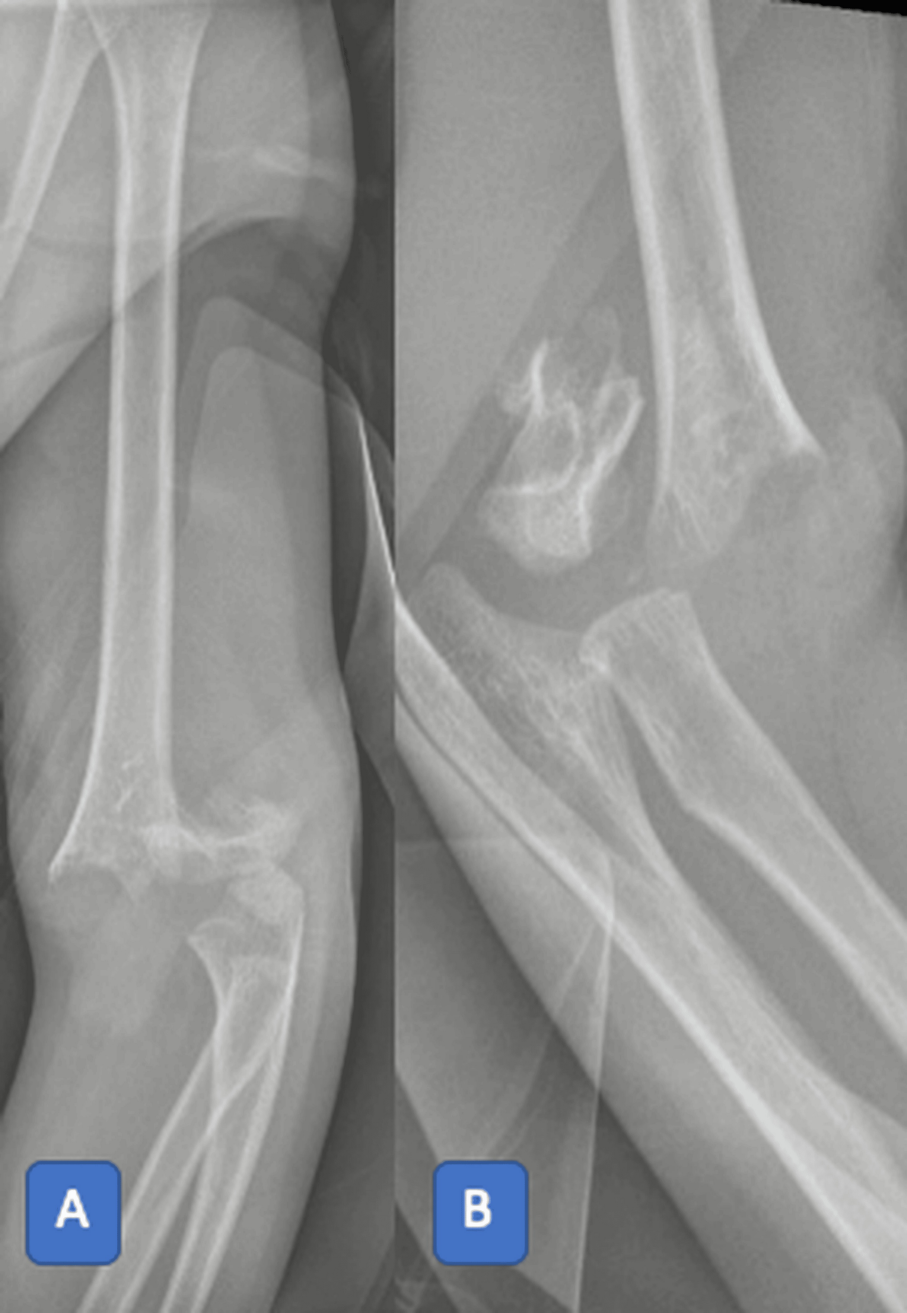
Anteroposterior (A) and lateral (B) radiographs demonstrating a displaced Gartland type III supracondylar fracture of the left humerus with posterior displacement of the distal fragment.

A four-year-old boy presented to the emergency department approximately 90 minutes after falling from a climbing frame and sustaining trauma to his left elbow. Clinical examination demonstrated marked swelling, visible deformity, and painful restriction of elbow motion. Neurovascular assessment revealed a pale and cold left hand with delayed capillary refill (>3 seconds). Radial and ulnar pulses were weak on palpation and difficult to detect by Doppler examination. Motor and sensory examination demonstrated no major neurological deficit at presentation. Radiographs obtained in anteroposterior and lateral views confirmed a displaced Gartland type III supracondylar humerus fracture with posterior displacement of the distal fragment (Figure 1).

Because of the unstable fracture configuration and concern for evolving vascular compromise, urgent operative management was indicated. External fixation using an external fixation system (DePuy Synthes, West Chester, PA, USA) was selected instead of standard Kirschner wire fixation to provide stable fracture alignment while simultaneously allowing unrestricted vascular access for possible exploration and repair. Following fracture stabilization, radial and ulnar pulses became completely non-palpable, and the hand remained pale and cold with persistent delayed capillary refill, raising concern for critical limb ischemia.

Immediate vascular exploration was therefore performed. Intraoperatively, the brachial artery was anatomically intact without evidence of transection. However, vascular compromise involving the proximal radial and ulnar arteries distal to the brachial bifurcation was identified and was presumed to be multifactorial, likely related to intimal injury with associated thrombotic occlusion and vascular spasm. Thrombectomy and selective catheterization using a Fogarty arterial embolectomy catheter (Edwards Lifesciences, Irvine, CA, USA) were successfully performed, resulting in immediate restoration of distal perfusion and palpable radial and ulnar pulses. Intraoperative Doppler sonographic evaluation using a GE ultrasound system (GE HealthCare, Chicago, IL, USA) confirmed normal arterial flow within the brachial, radial, and ulnar arteries with adequate hand perfusion. Total time from injury to vascular restoration was approximately four hours.

Postoperatively, the patient received individualized weight-adjusted rivaroxaban therapy (Xarelto®; Bayer AG, Leverkusen, Germany) for three months following interdisciplinary consultation between pediatric orthopedic and vascular surgical teams. The rationale for anticoagulation was to prevent postoperative thrombosis after vascular intervention, although standardized pediatric protocols remain lacking. No bleeding complications occurred during treatment.

Follow-up Doppler ultrasonography at six months demonstrated preserved arterial patency and intact distal perfusion without evidence of recurrent thrombosis. Clinical follow-up confirmed a full range of motion, preserved grip strength, intact motor and sensory function, and complete recovery of hand function without vascular or neurological deficits.

Case 2

**Figure 2:**
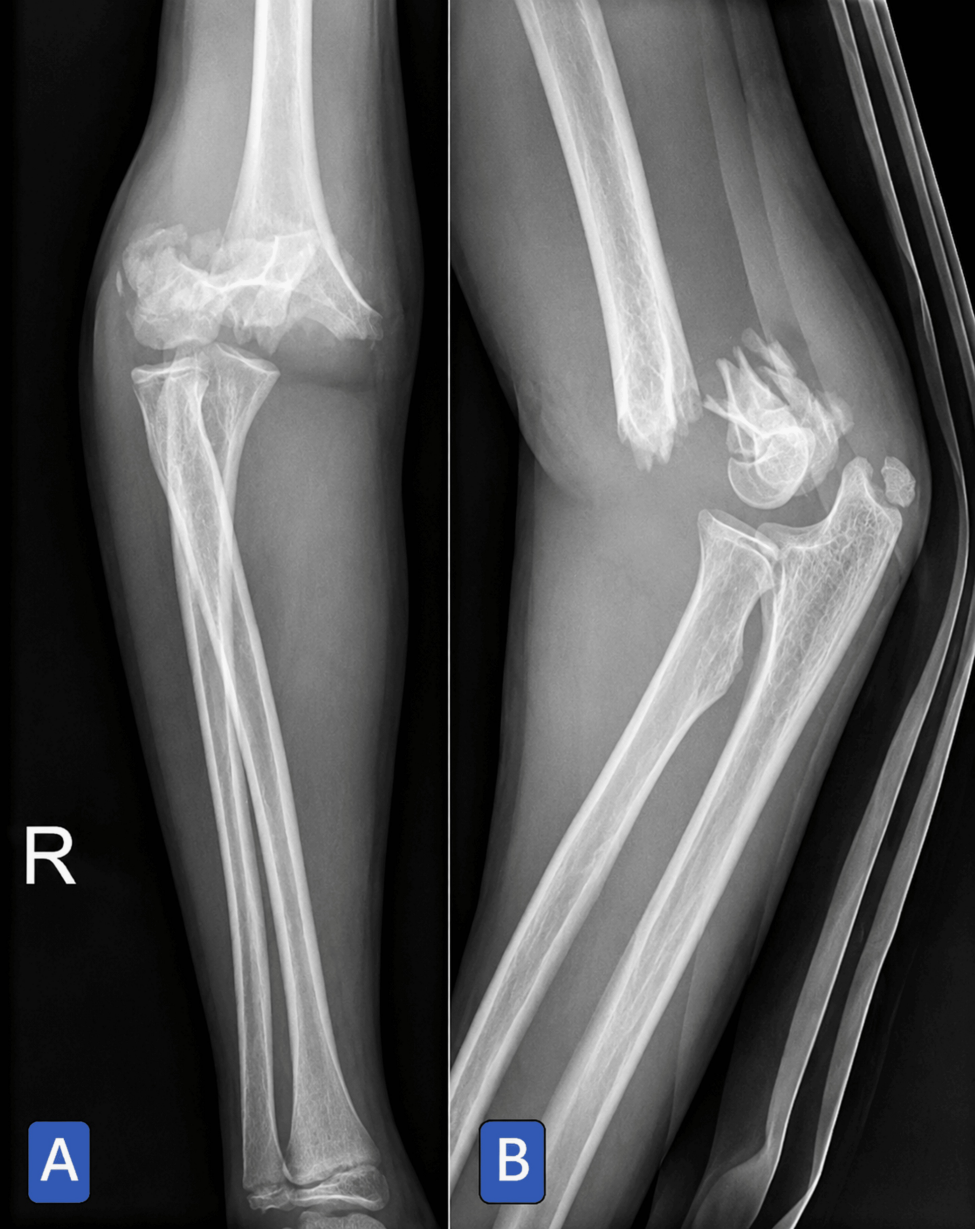
Anteroposterior (A) and lateral (B) radiographs demonstrating a displaced Gartland type III supracondylar fracture of the right humerus with posterior displacement of the distal fragment.

An 11-year-old girl presented to the emergency department approximately two hours after falling from a trampoline and sustaining trauma to her right elbow. Clinical examination revealed substantial swelling, deformity, and painful limitation of elbow motion. Neurovascular assessment demonstrated a pale right hand with delayed capillary refill and absent palpable radial and ulnar pulses. Doppler examination revealed markedly reduced distal arterial signals. Sensory examination demonstrated numbness of the hand without a persistent motor deficit. Radiographic evaluation with anteroposterior and lateral views confirmed a displaced Gartland type III supracondylar humerus fracture with posterior displacement of the distal fragment (Figure 2).

Given the clinical signs of acute vascular compromise, urgent surgical treatment was undertaken without delaying intervention for advanced vascular imaging. External fixation using an external fixation system (DePuy Synthes, West Chester, PA, USA) was selected because it allowed stable fracture reduction while simultaneously minimizing additional soft tissue manipulation and preserving unrestricted access for vascular exploration and microsurgical repair.

During surgical exploration, the brachial artery was found entrapped directly within the fracture site. Careful microsurgical release of the artery followed by primary vascular repair was successfully performed, resulting in immediate restoration of distal perfusion and palpable peripheral pulses. Intraoperative Doppler sonographic evaluation using a GE ultrasound system (GE HealthCare, Chicago, IL, USA) confirmed normal blood flow through the brachial, radial, and ulnar arteries with adequate hand perfusion. Total ischemia time from injury to vascular restoration was approximately five hours.

Postoperatively, the patient received individualized weight-adjusted rivaroxaban therapy (Xarelto®; Bayer AG, Leverkusen, Germany) for three months after interdisciplinary evaluation by pediatric orthopedic and vascular surgical teams. The decision for postoperative anticoagulation was based on concern for thrombotic complications following vascular repair, despite limited pediatric evidence and off-label use considerations. No anticoagulation-related complications were observed.

Sensory symptoms resolved completely during follow-up. Doppler ultrasonography performed during follow-up demonstrated maintained arterial patency without recurrent vascular compromise. At six-month clinical follow-up, the patient demonstrated preserved elbow function, intact motor and sensory function, normal distal perfusion, and complete recovery without vascular or neurological sequelae.

## Discussion

Vascular injury associated with pediatric supracondylar humerus fractures is uncommon but represents a potentially limb-threatening complication requiring rapid recognition and urgent management [1,2]. Displaced Gartland type III and IV fractures are particularly associated with neurovascular compromise because of hyperextension injury, posterior fragment displacement, arterial entrapment, intimal injury, thrombosis, or vasospasm involving the brachial artery and its distal branches [3-5]. Early diagnosis and restoration of distal perfusion are essential to prevent prolonged ischemia, compartment syndrome, neurological injury, and long-term functional impairment.

Clinical neurovascular assessment remains the cornerstone of diagnosis. Careful evaluation of brachial, radial, and ulnar pulses, capillary refill, skin temperature, hand color, Doppler signals, and motor-sensory function is mandatory in all displaced supracondylar fractures [6,7]. Particular attention should be paid to distinguishing between the “pink pulseless hand” and the “pale pulseless hand.” While some patients with preserved perfusion after fracture reduction may be managed with close observation, a pale, cold, pulseless extremity generally indicates critical ischemia and requires urgent vascular exploration [6,7].

Although Doppler ultrasonography and CT angiography may assist in selected patients, advanced vascular imaging should not delay operative intervention when clinical signs strongly suggest limb-threatening vascular compromise. In both presented cases, the decision for urgent surgery was based primarily on clinical findings and evolving ischemia rather than preoperative angiographic imaging. Intraoperative Doppler sonography was subsequently used to confirm restoration of arterial flow following vascular intervention.

The brachial artery is the vessel most commonly involved in these injuries because of its close anatomical relationship to the distal humerus [3-5]. In Case 1, the brachial artery remained anatomically intact, whereas distal vascular compromise involving the radial and ulnar arteries was presumed secondary to intimal injury with associated thrombosis and vasospasm distal to the brachial bifurcation. In Case 2, the brachial artery was directly entrapped within the fracture site and required microsurgical release and primary vascular repair. In both cases, rapid vascular restoration was achieved within several hours after injury, likely contributing to the favorable neurological and functional outcomes observed during follow-up.

Closed reduction with percutaneous Kirschner wire fixation remains the standard treatment for most displaced pediatric supracondylar humerus fractures [1-3]. However, in selected cases with associated vascular injury, external fixation may offer important advantages. In the present cases, external fixation provided stable fracture alignment while minimizing repeated manipulation and simultaneously preserving unrestricted access for vascular exploration and repair. This approach facilitated interdisciplinary management between pediatric orthopedic and vascular surgical teams while avoiding additional soft tissue trauma during vascular reconstruction.

The optimal postoperative anticoagulation strategy following pediatric vascular repair remains incompletely defined because prospective pediatric data are limited and standardized treatment protocols are lacking. Although rivaroxaban use in children remains off-label in many clinical settings, emerging evidence suggests that carefully monitored, weight-adjusted anticoagulation may be considered in selected cases after vascular intervention [8]. In our patients, individualized postoperative rivaroxaban therapy was selected after interdisciplinary discussion with the aim of reducing thrombotic risk following vascular repair. No bleeding complications or recurrent vascular compromise occurred during treatment or follow-up. Nevertheless, the limited evidence base and small number of reported cases require cautious interpretation, and further studies are needed to establish evidence-based pediatric anticoagulation recommendations after traumatic vascular injury.

These cases further emphasize the importance of interdisciplinary collaboration among pediatric orthopedic surgeons, vascular surgeons, anesthesiologists, and radiologists in the management of complex supracondylar humerus fractures with vascular compromise. Early recognition, prompt fracture stabilization, timely vascular exploration, and close postoperative monitoring remain essential for optimizing vascular and functional outcomes in affected children.

## Conclusions

Pediatric supracondylar humerus fractures with vascular compromise represent rare but potentially limb-threatening injuries requiring rapid diagnosis and urgent interdisciplinary management. Careful neurovascular assessment, prompt fracture stabilization, and timely vascular exploration are critical to restoring distal perfusion and preventing ischemic complications. In the presented cases, external fixation combined with vascular intervention resulted in favorable vascular and functional recovery without long-term neurological deficits. Postoperative weight-adjusted rivaroxaban therapy was used as part of individualized management following vascular repair without observed thrombotic or bleeding complications; however, evidence regarding postoperative anticoagulation after pediatric vascular trauma remains limited, and standardized treatment protocols are currently lacking. Further studies are required to clarify optimal postoperative management strategies and long-term outcomes in pediatric supracondylar humerus fractures complicated by vascular injury.
